# Uptake and determinants of HPV vaccination in South Asia: a systematic review and meta-analysis

**DOI:** 10.3389/fpubh.2024.1453704

**Published:** 2024-12-11

**Authors:** Khola Noreen, Samina Naeem Khalid, Manal Abdulaziz Murad, Mukhtiar Baig, Shahzad Ali Khan

**Affiliations:** ^1^Department of Community Medicine, Rawalpindi Medical University, Rawalpindi, Pakistan; ^2^Health Services Academy, Islamabad, Islamabad, Pakistan; ^3^Department of Family and Community Medicine, Faculty of Medicine in Rabigh, King Abdulaziz University, Jeddah, Saudi Arabia; ^4^Department of Biochemistry, Faculty of Medicine in Rabigh, King Abdulaziz University, Jeddah, Saudi Arabia

**Keywords:** HPV vaccination, vaccination determinants, cervical cancer, South Asia, meta-analysis

## Abstract

**Background:**

Cervical cancer burden in South Asia is among the highest globally. Due to the lack of national immunization programs, the prevalence of human papillomavirus (HPV) infection and vaccine uptake remains unknown. This systematic review and meta-analysis aim to determine the prevalence of HPV vaccine uptake in South Asia.

**Methods:**

We conducted a comprehensive search of MEDLINE (via PubMed), Embase, the Cochrane Library, and the Web of Science, covering the period from inception to May 20, 2024. We included observational studies reporting HPV vaccine uptake in South Asia, without any language filters or restrictions. The search strategy involved MeSH terms and relevant keywords related to “Papillomavirus Infections,” “Vaccination,” and “Uptake.” MetaXL and STATA were used to perform a proportional meta-analysis and meta-regression analysis.

**Results:**

Out of 3,913 articles identified, 17 articles (10,585 participants) were included in the systematic review. The pooled prevalence of vaccine uptake was 8% (95% CI 1–21). There was high heterogeneity between studies (*I*^2^ = 100%). The pooled prevalence of adequate knowledge of the HPV vaccine was 41% (95% CI 28–55, *I*^2^ = 99%). The pooled prevalence of a favorable attitude toward the HPV vaccine was 56% (95% CI 47–66, *I*^2^ = 98%). In the univariate meta-regression model, good knowledge significantly predicted HPV vaccine uptake (*p* = 0.003), while no covariates were found to be significant predictors of attitudes toward HPV vaccine uptake.

**Conclusion:**

The findings of this meta-analysis indicate a low pooled prevalence of HPV vaccine uptake (8%) in South Asian countries. The pooled prevalence of adequate knowledge and a favorable attitude toward the vaccine were 41 and 56%, respectively. In the univariate meta-regression model, knowledge of the HPV vaccine uptake was the only significant predictor of vaccine uptake.

**Systematic review registration:**

Systematic review is registered at Prospero through the link https://www.crd.york.ac.uk/prospero/display_record.php?ID=CRD42024547393

## Introduction

According to the GLOBOCAN 2020 database, there were over six million new cases and 340,000 deaths attributed to cervical cancer in 2020, making up nearly 3 % of both global cancer incidence and mortality (1). Cervical cancer is the fourth most common cancer in women, following breast, colorectal, and lung cancers. Low and middle-income countries carry the largest burden, accounting for almost 80% of new cases and 90% of deaths from cervical cancer (2).

Several risk factors have been identified for cervical cancer, including tobacco use, immunodeficiency, multiple pregnancies, and family history. However, the primary cause is widely accepted to be human papillomavirus (HPV) infection (3–5). HPV is one of the most prevalent sexually transmitted diseases in both men and women (6). There are over 130 serotypes of HPV, with high-risk serotypes 16 and 18 being most commonly associated with cervical cancer (7). The central role of HPV in cervical cancer pathogenesis makes this disease highly preventable.

The introduction of HPV vaccines, such as Gardasil and Cervarix, represents a groundbreaking advancement in the prevention of cervical cancer and other HPV-related diseases (8, 9). These vaccines offer long-term immunity with high efficacy but have potential side effects and require multiple doses. Following promising clinical trial results, 64 countries have launched national immunization programs with the goal of vaccinating 90% of girls by age 15 by 2030 (10, 11). The widespread rollout of the HPV vaccine in most developed countries has shifted the global burden of the disease toward low and middle-income countries (12). Currently, 85% of high-income countries, 25% of upper-middle-income countries, 30% of lower-middle-income countries, and 60% of low-income countries have national immunization programs (10).

The South Asian region, which includes India, Pakistan, Bangladesh, Nepal, Afghanistan, Sri Lanka, Bhutan, and Maldives, is home to 21% of the world’s population (13). Most countries in this region fall under the “low-middle-income country (LMIC)” classification. Despite the World Health Organization (WHO) highlighting strategies for cervical cancer eradication in LMICs, the uptake of the HPV vaccine in South Asia has been inadequate, with India alone accounting for a fifth of the global burden of the disease (10). Only Bhutan, Sri Lanka, and Maldives have included HPV vaccination in their national immunization programs (14).

Some progress has been made recently, with the completion of pilot projects for a vaccination program in Bangladesh and the launch of India’s first locally produced HPV vaccine (15). However, several sociocultural factors pose significant obstacles to the rapid rollout of vaccination programs across the region (16, 17). Data on the uptake of the HPV vaccine and attitudes toward vaccination are scarce and inconclusive. Therefore, systematic review is conducted and proportional meta-analysis to determine the pooled prevalence of HPV vaccine uptake and its determinants in South Asia.

## Methods

The present systematic review and meta-analysis followed the guidelines of the Preferred Reporting Items for Systematic Reviews and Meta-Analyses (PRISMA) and was registered with PROSPERO (CRD42024547393) (18).

### Search strategy

MEDLINE (via PubMed), Embase, the Cochrane Library, and the Web of Science from inception to May 20, 2024, without any language filters or restrictions were searched. The search strategy included MeSH terms and relevant keywords for “Papillomavirus Infections,” “Vaccination,” and “Uptake.” Detailed search strategy include Boolean operators ‘OR’ and ‘AND’ (“Papillomavirus Infections” [Mesh] OR Papillomavirus Infection OR Human Papillomavirus Infection OR Human Papillomavirus Infections OR HPV Infection OR HPV Infections) AND (“Vaccination” [Mesh] OR Vaccinations OR Active Immunization OR Active Immunizations) AND (“Uptake” [All Fields] OR “Practice” [All Fields] OR “utilization” [All Fields]). We also hand-searched the reference lists of relevant articles for additional references.

(“Papillomavirus” [Mesh] OR Human Papillomavirus OR Human Papillomavirus OR HPV) AND (“Vaccination” [Mesh] OR Vaccinations OR Active Immunization OR Active Immunizations) AND (“Uptake” [All Fields] OR “Practice” [All Fields] OR “utilization” [All Fields]) AND (India OR Pakistan OR Bangladesh OR Nepal OR Afghanistan OR Sri Lanka OR Bhutan OR Maldives).

### Study selection and eligibility criteria

The search strategy imported the references into Endnote version 20. Two authors (KN and SN) independently screened the articles, with disagreements resolved by other authors (MAM, MBM, and SAK). The eligibility requirements were as follows: (1) The population consists of South Asian nationals who have not received a cervical cancer diagnosis, (2) the outcome is the prevalence of HPV vaccine uptake, and (3) the study design is observational studies.

### Data extraction and outcomes

Data was extracted data on study characteristics (author name, publication year, study setting, and sample size), participant sociodemographic characteristics (age, sex, marital status, literacy, and employment), and our outcomes using Microsoft Excel. The primary outcome of interest was the prevalence of HPV vaccine uptake. The secondary outcomes were the prevalence of good knowledge of HPV vaccination and a positive attitude toward HPV vaccination. Two authors (KN and SN) independently extracted data, and disagreements were resolved by other authors (MAM, MB, and SAK).

### Quality assessment

The “National Institute of Health (NIH) Quality Assessment Tool for Observational Cohort and Cross-sectional Studies” was used for quality assessment (19). Two authors (KN and SN) independently assessed the quality of the included studies, with disagreements settled by a third author (MAM, MB, and SAK). The traffic light plots was constructed using Robvis (20).

### Data analysis

As all outcomes were prevalence, we performed a meta-analysis of proportions. The Freeman-Tukey double arcsine transformation was used to stabilize variances. The random-effects model with the DerSimonian Laird method to pool transformed proportions before back-transforming them. The forest plots and computed *I*^2^ statistics were constructed to assess heterogeneity. The subgroup analysis was conducted based on the country of study and occupation. Meta-regression was also performed to determine predictors of the prevalence of HPV vaccine uptake and attitude toward the HPV vaccine. All statistical analyses were performed in MetaXL and STATA.

## Results

### Search results

**Figure 1:**
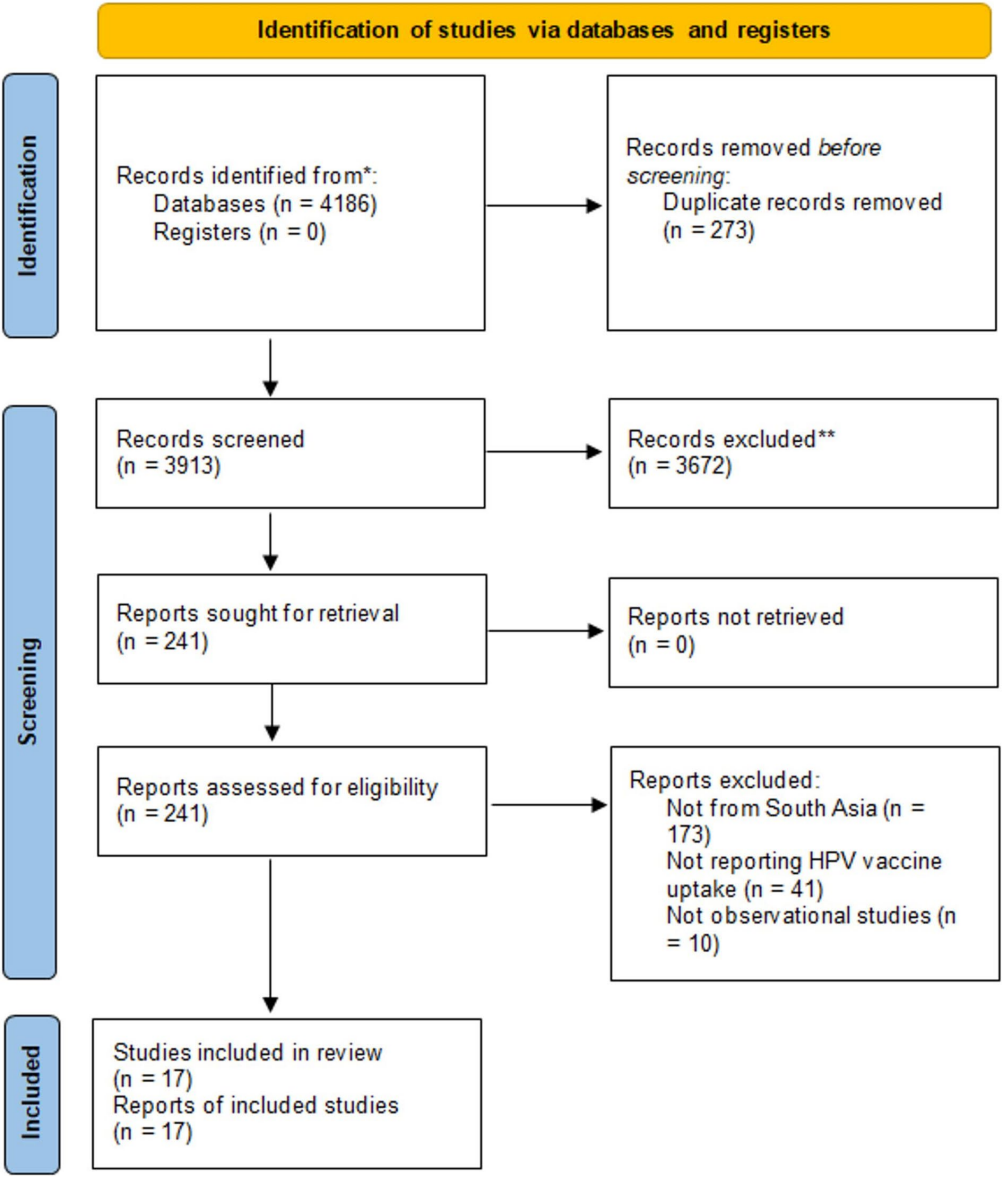
PRISMA flowchart.

The initial search retrieved 4,186 articles. After removing 273 duplicates, we assessed the remaining 3,913 articles for eligibility. The titles and abstracts were reviewed and excluded 3,672 articles. Full texts of the remaining 241 articles were assessed against the eligibility criteria. Finally, 17 articles were included. Figure 1 provides details of the screening process.

### Study characteristics

Of the 17 studies included in our review, 8 were conducted in India (21–28), 5 in Pakistan (29–33), 3 in Bangladesh (34–36), and 1 in Bhutan (37). The publication years ranged from 2015 to 2024. A total of 10,585 participants were included, with 8,413 (79.5%) being females. The mean age ranged from 19 to 40 years. Table 1 summarizes the characteristics of the included studies.

**Table 1:** Characteristics of included studies.

Author	Year	Country	Sample size	Mean Age, in years (S.D.)	Female, *N* (%)	Married, *N* (%)	Education illiterate/primary/secondary/higher	Occupation, *N* (%)
Shamsi	2024	Pakistan	480	27.4 (10.7)	367 (77)	135 (28)	0/0/0/480	Unemployed 346 (72) Employed 134 (28)
Shafiq	2023	Pakistan	250	28.9 (7.99)	250 (100)	124 (50)	NR	Healthcare 250 (100)
Qayum	2022	Bangladesh	400	NR	400 (100)	0 (0)	144/111/145/NR	Employed 400 (100)
Qayum	2021	Bangladesh	956	41.23 (9.09)	256 (100)	845 (88)	230/696/30/NR	Unemployed 928 (97) Employed 28 (3)
Chellapandian	2021	India	318	NR	247 (78)	86 (27)	0/0/0/318	Healthcare 318 (100)
Baussano	2021	Bhutan	909	19 (17–22)*	909 (100)	NR	NR	NR
Jacob	2021	India	403	NR	403 (100)	NR	NR	NR
Sharma	2021	India	397	40 (34–49)*	397 (100)	338 (85)	0/0/0/397	Employed 397 (100)
Banik	2020	Bangladesh	600	36.8 (2.0)	600 (100)	377 (63)	NR	Unemployed 274 (46) Employed 326 (54)
Rehman	2020	India	1,020	NR	1,020 (100)	905 (89)	131/166/171/152	Unemployed 880 (86) Employed 140 (14)
Hirania	2020	Pakistan	384	30 (7.6)	384 (100)	266 (69.2)	1/38/70/271	Unemployed 270 (70) Employed 114 (30)
Padmanabha	2019	India	263	20.66 (1.12)	263 (100)	NR	0/0/0/263	Healthcare 263 (100)
Shaikh	2019	Pakistan	400	22.1 (2.1)	280 (400)	63 (16)	NR	Unemployed 300 (75) Employed 100 (25)
Sharma	2019	India	230	19.8 (1.94)	230 (100)	NR	0/0/0/230	NR
Zaheer	2017	Pakistan	1,038	20.62 (1.54)	1,038 (100)	39 (3.76)	NR	NR
Rashid	2016	India	1,580	NR	684 (43)	NR	0/0/0/1580	NR
Swarnapriya	2015	India	957	19.25 (1.64)	685 (71)	NR	0/0/0/957	Healthcare 957 (100)

*Median age, in years (Interquartile range); N, Number; NR, Not reported.

### Quality assessment of included studies

**Figure 2:**
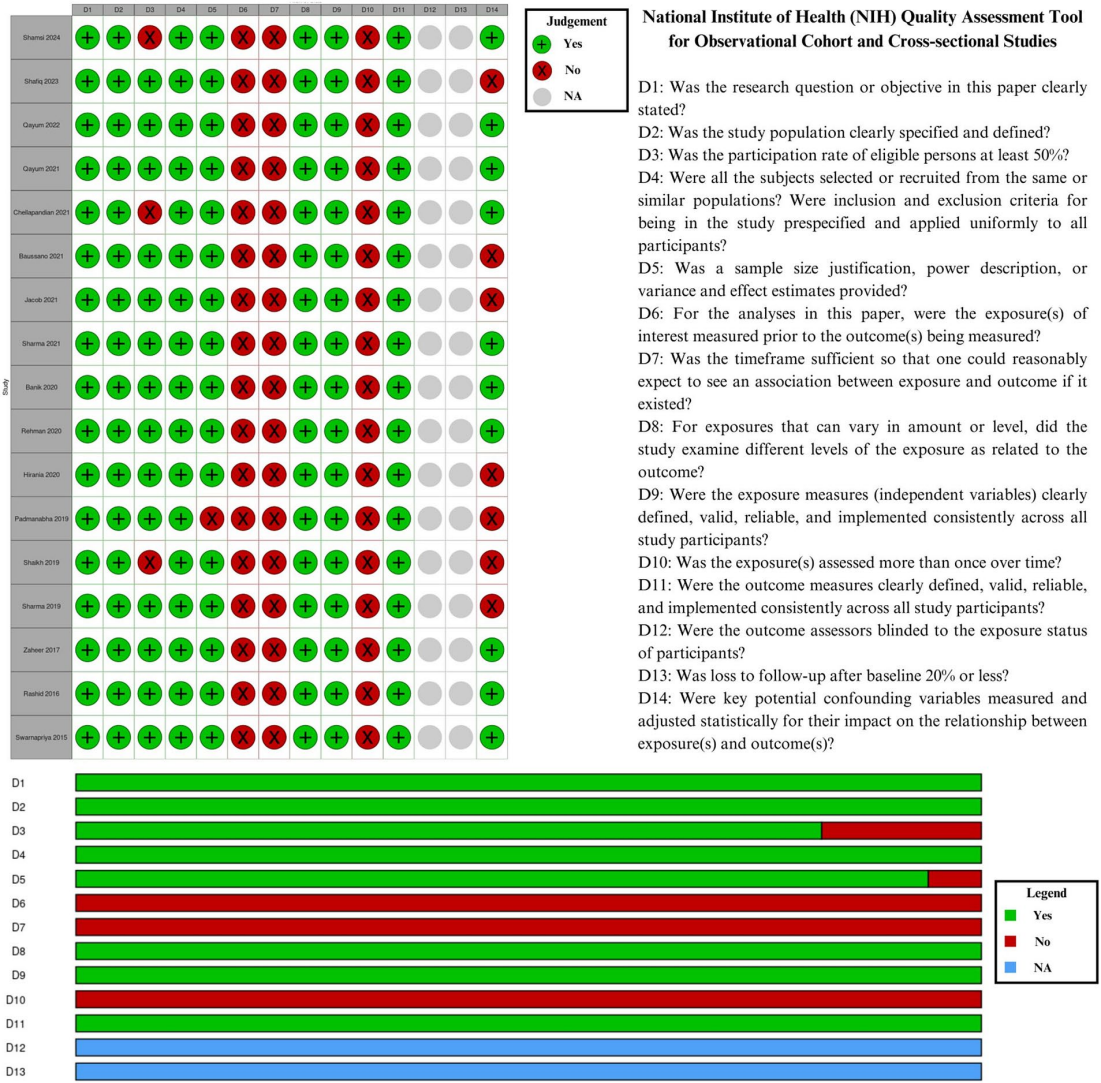
Quality assessment of included studies.

In Figure 2, the quality assessment of the included studies was presented as a traffic light plot and a summary plot. Due to their cross-sectional nature, all studies scored poorly in D6 (exposures measured before outcomes), D7 (sufficient timeframe), and D10 (exposures assessed over time). D12 (blinding) and D13 (loss to follow-up) were considered not applicable exclusively to cohort studies.

### Proportional meta-analysis for vaccine uptake

**Figure 3:**
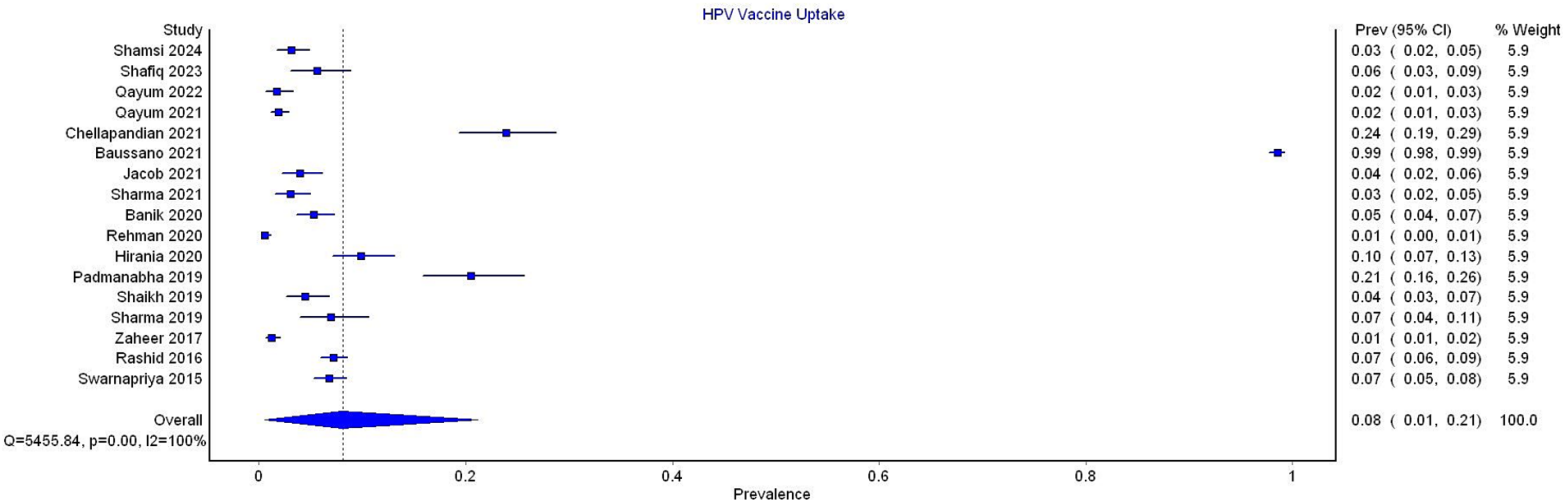
Forest plot for the prevalence of HPV vaccine uptake.

All 17 studies reported data on HPV vaccine uptake. The pooled prevalence of vaccine uptake was 8% (95% CI 1–21). The substantial heterogeneity was noted (*I*^2^ = 100%) (Figure 3).

**Figure 4:**
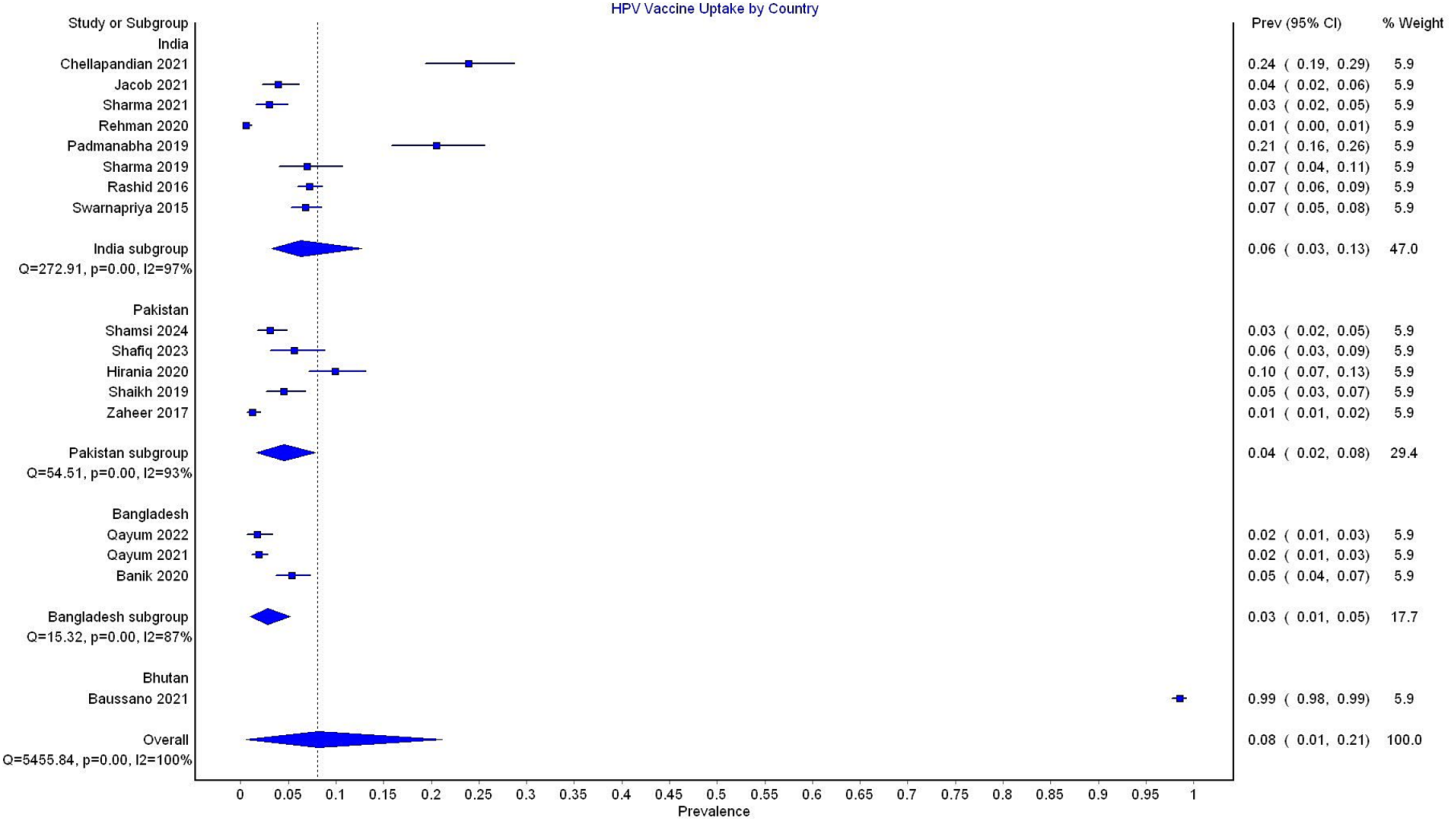
Forest plot depicting subgroup analysis by country for the prevalence of HPV vaccine uptake.

In the subgroup analysis by country, the pooled prevalence was 6% (95% CI 3–13, 8 studies, *I*^2^ = 97%) for India, 4% (95% CI 2–8, 5 studies, *I*^2^ = 93%) for Pakistan, and 3% (95% CI 1–5%, 3 studies, *I*^2^ = 87%) for Bangladesh. Only one study reported data from Bhutan, which showed a prevalence of 99% (Figure 4).

**Figure 5:**
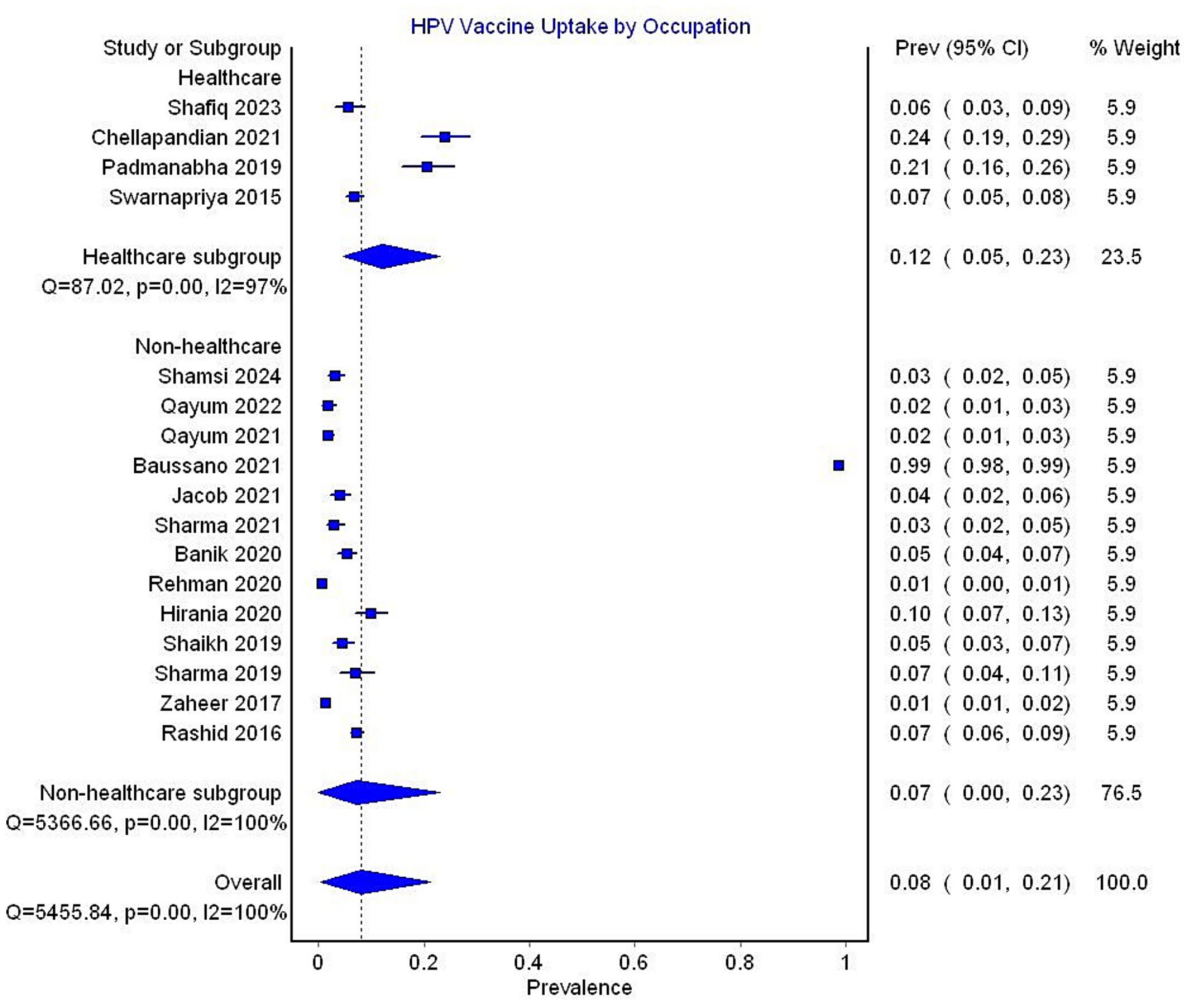
Forest plot depicting subgroup analysis by occupation for the prevalence of HPV vaccine uptake.

In the subgroup analysis by occupation, studies reporting data exclusively on healthcare workers reported a pooled prevalence of 12% (95% CI 5–23, 4 studies, *I*^2^ = 97%), compared to a prevalence of 7% (95% CI 0–23, 13 studies, *I*^2^ = 100%) reported in studies not exclusively focusing on healthcare workers (Figure 5).

### Proportional meta-analysis for knowledge of HPV vaccine

**Figure 6:**
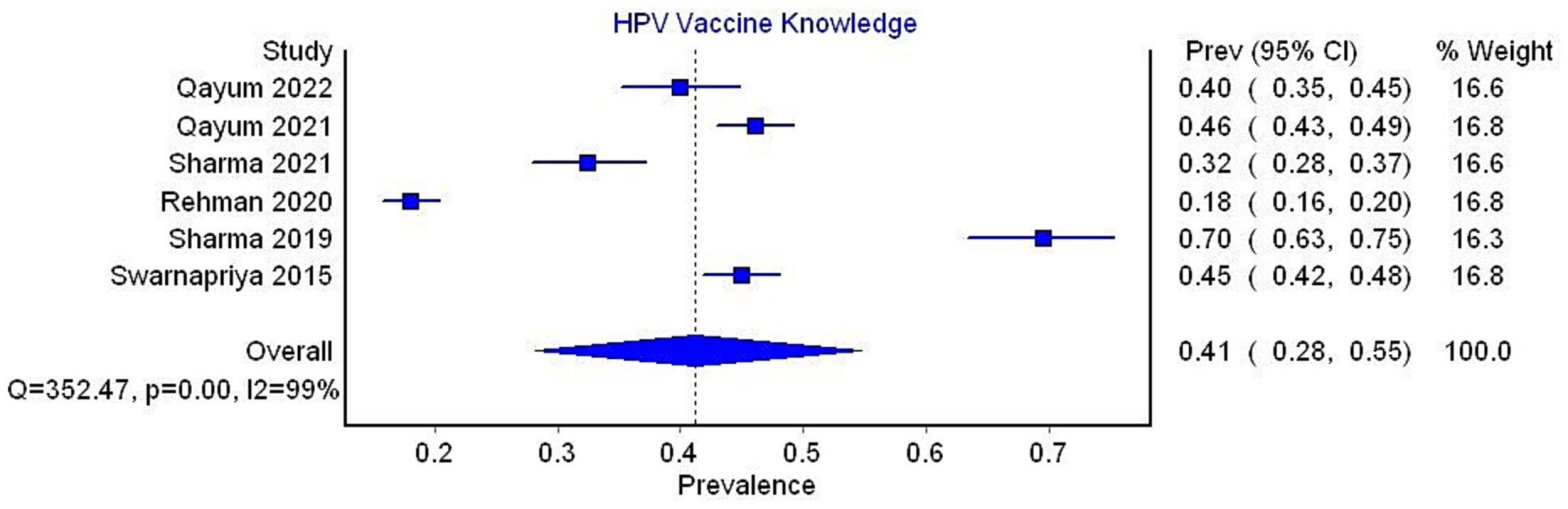
Forest plot for the prevalence of HPV vaccine knowledge.

The pooled prevalence of adequate knowledge of the HPV vaccine was 41% (95% CI 28–55, *I*^2^ = 99%), based on data from 6 studies (Figure 6).

**Figure 7:**
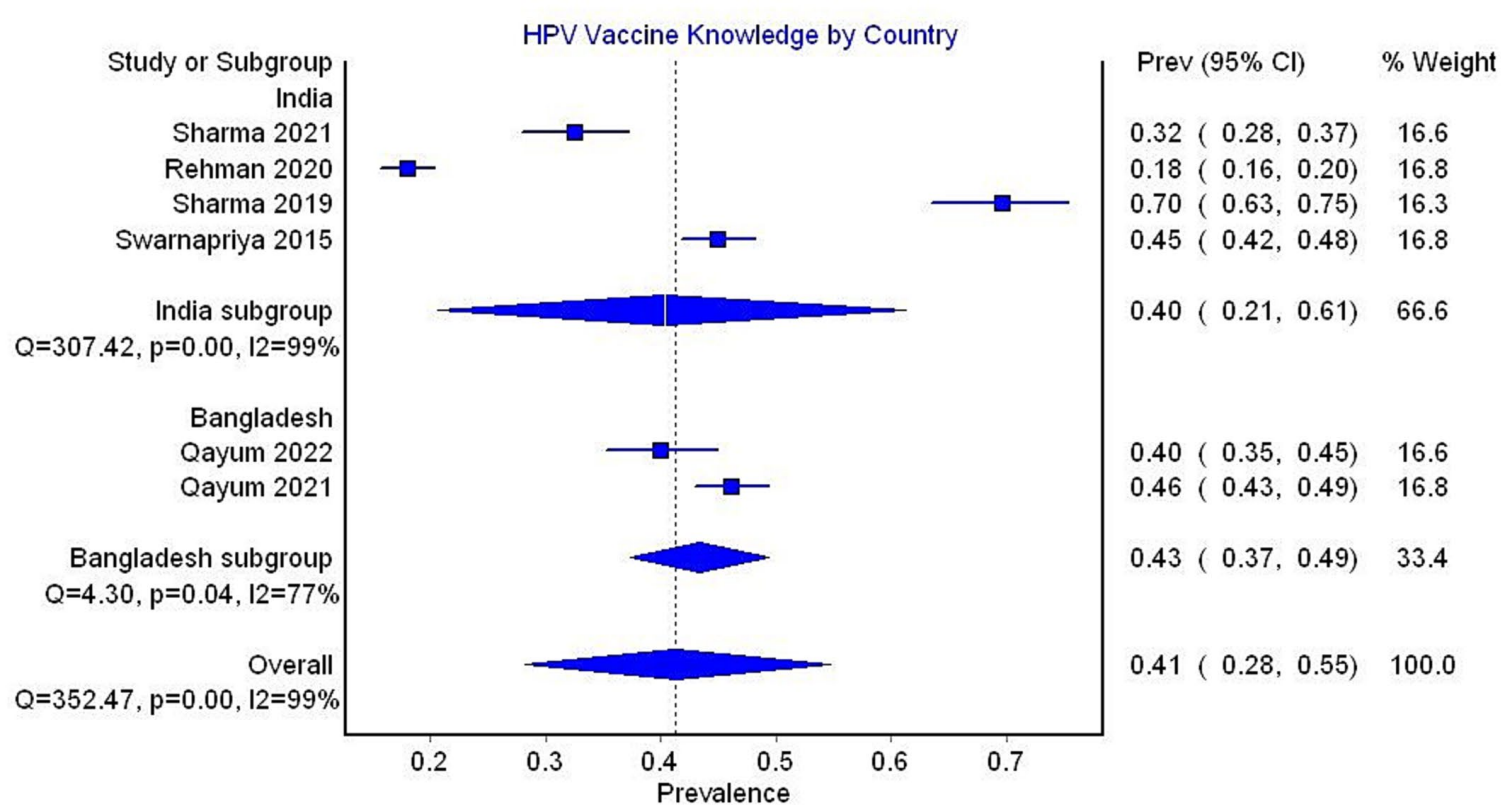
Forest plot depicting subgroup analysis by country for the prevalence of HPV vaccine knowledge.

In the subgroup analysis by country, HPV vaccine knowledge was reported to be 40% (95% CI 21–61, 4 studies, *I*^2^ = 99%) in India and 43% (95% CI 37–49, 2 studies, *I*^2^ = 77%) in Bangladesh (Figure 7).

**Figure 8:**
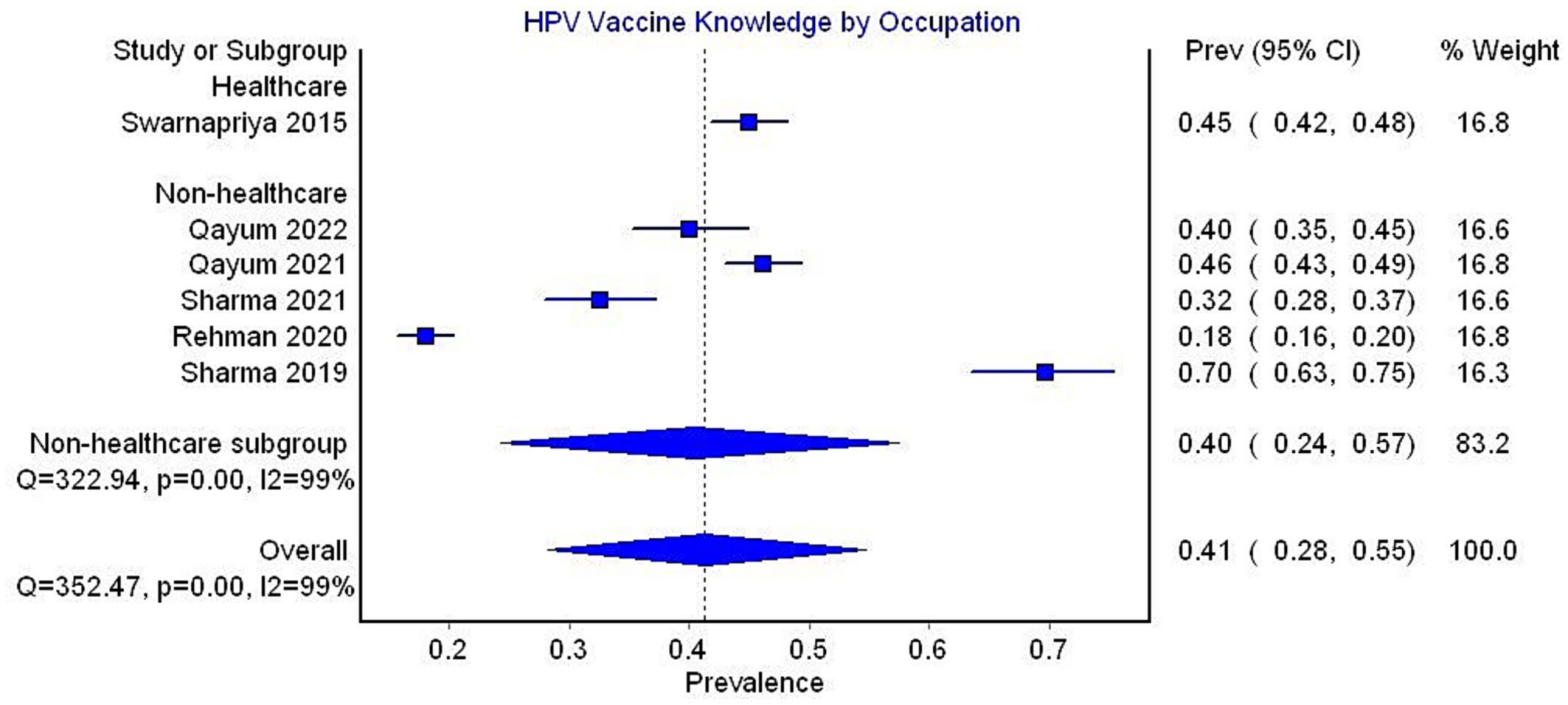
Forest plot depicting subgroup analysis by occupation for the prevalence of HPV vaccine knowledge.

In the subgroup analysis by occupation, only one study reporting data on healthcare professionals was included, with 45% of the sample having adequate knowledge. The pooled prevalence for non-healthcare studies was 40% (95% CI 24–57, 5 studies, *I*^2^ = 99%) (Figure 8).

### Proportional meta-analysis for attitudes toward HPV vaccine

**Figure 9:**
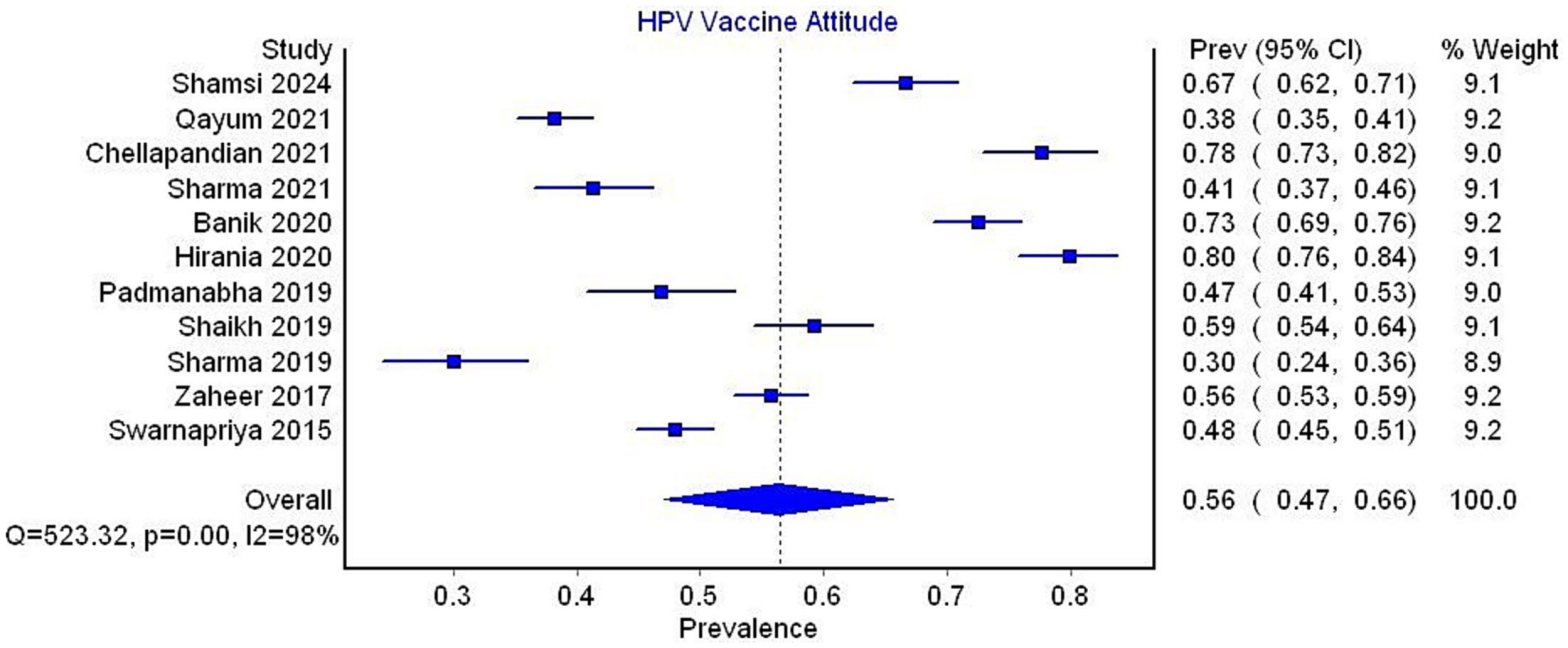
Forest plot for prevalence of attitude toward HPV vaccine.

Eleven studies were included in the analysis and found that the pooled prevalence of a favorable attitude toward the HPV vaccine was 56% (95% CI 47–66, *I*^2^ = 98%) (Figure 9).

**Figure 10:**
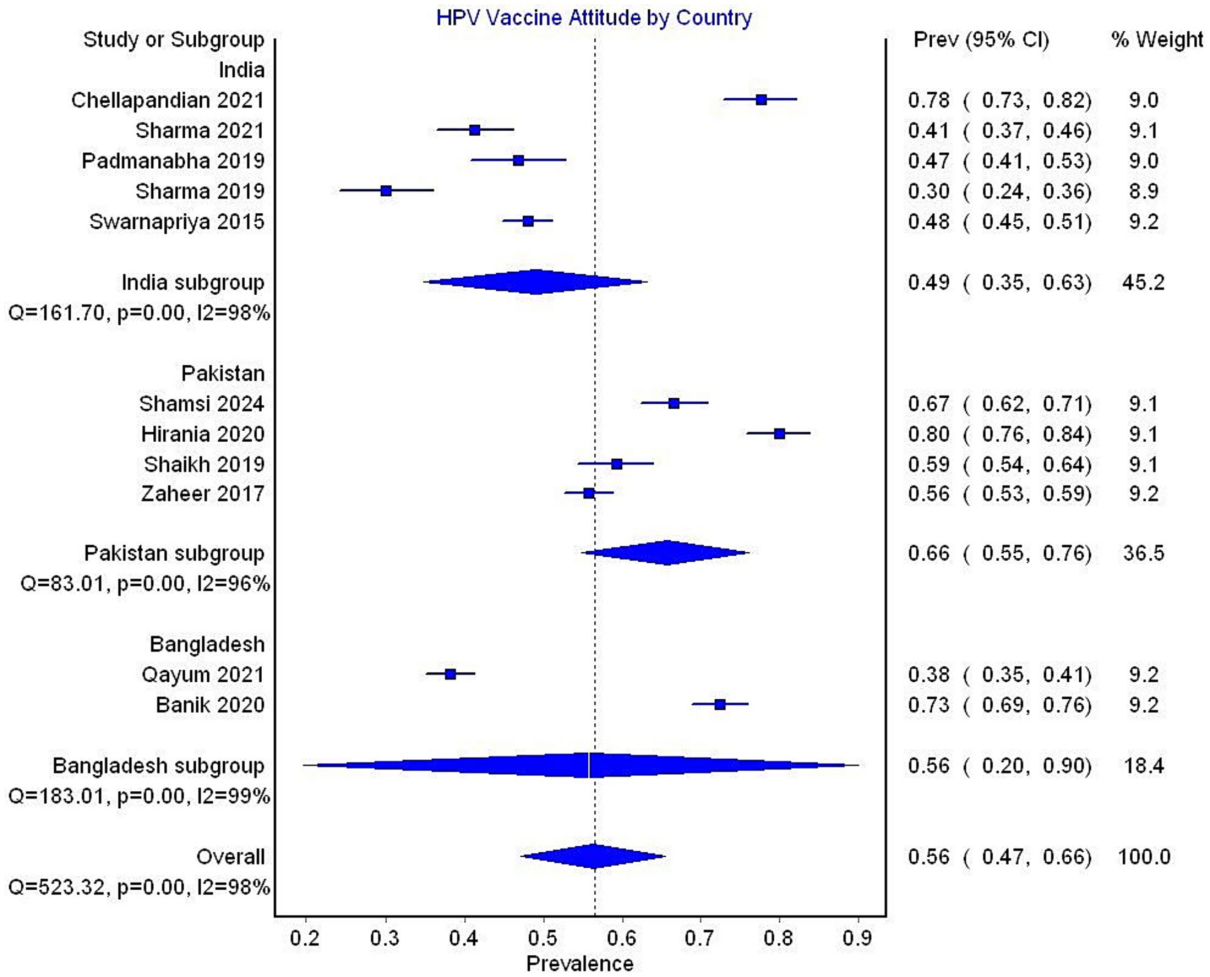
Forest plot depicting subgroup analysis by country for the prevalence of attitude toward HPV vaccine.

In the subgroup analysis by country, the pooled prevalence of a favorable attitude toward the HPV vaccine was 49% (95% CI 35–63, 5 studies, *I*^2^ = 98%) for India, 66% (95% CI 55–76, 4 studies, *I*^2^ = 96%) for Pakistan, and 56% (95% CI 20–90, 2 studies, *I*^2^ = 99%) for Bangladesh (Figure 10).

**Figure 11:**
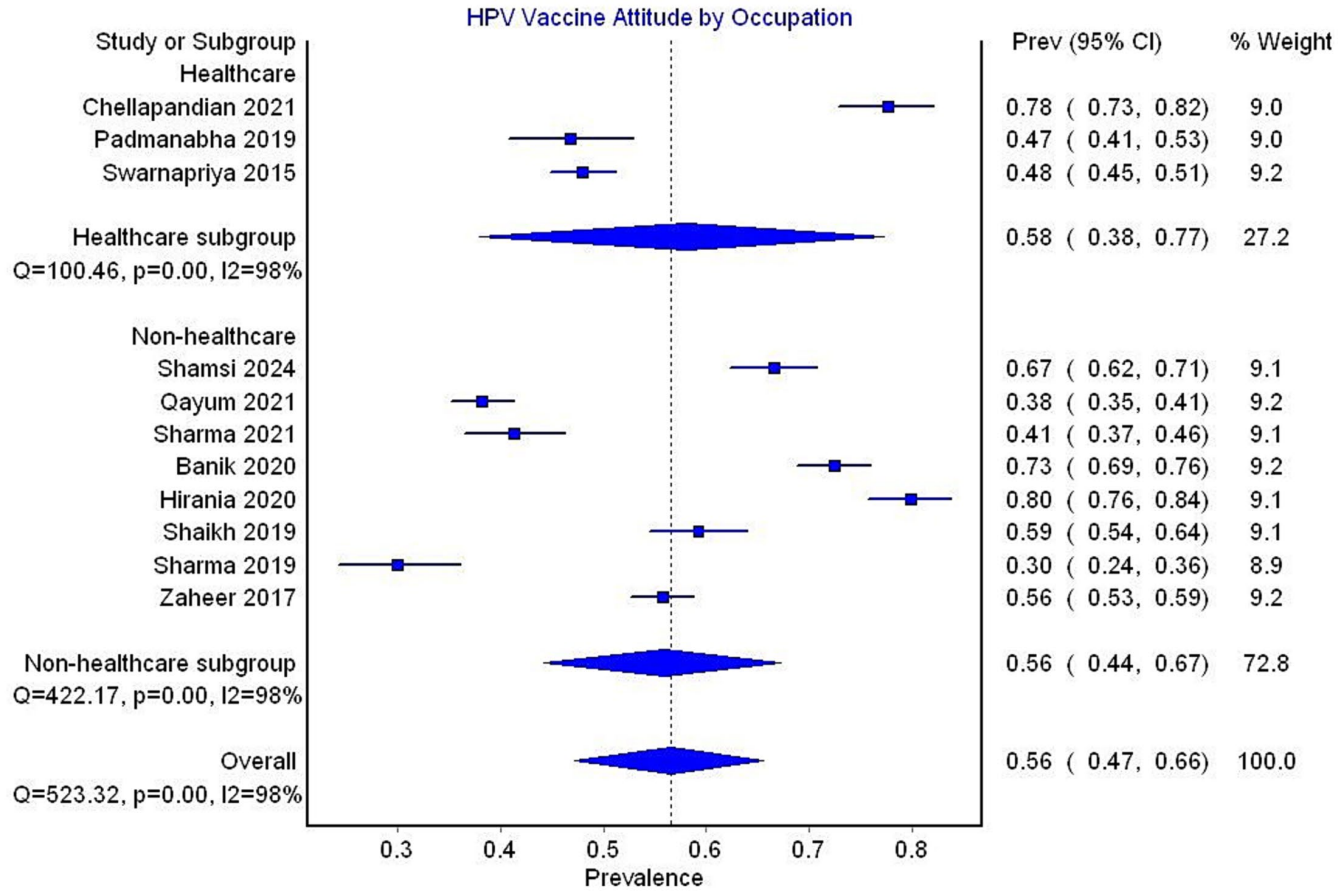
Forest plot depicting subgroup analysis by occupation for prevalence of attitude toward HPV vaccine.

In the subgroup analysis by occupation, studies exclusively reporting data on healthcare workers showed a pooled prevalence of a favorable attitude toward the HPV vaccine of 58% (95% CI 38–77, 3 studies, *I*^2^ = 98%), compared to a prevalence of 56% (95% CI 44–67, 8 studies, *I*^2^ = 98%) reported in studies that did not exclusively focus on healthcare workers (Figure 11).

**Figure 12:**
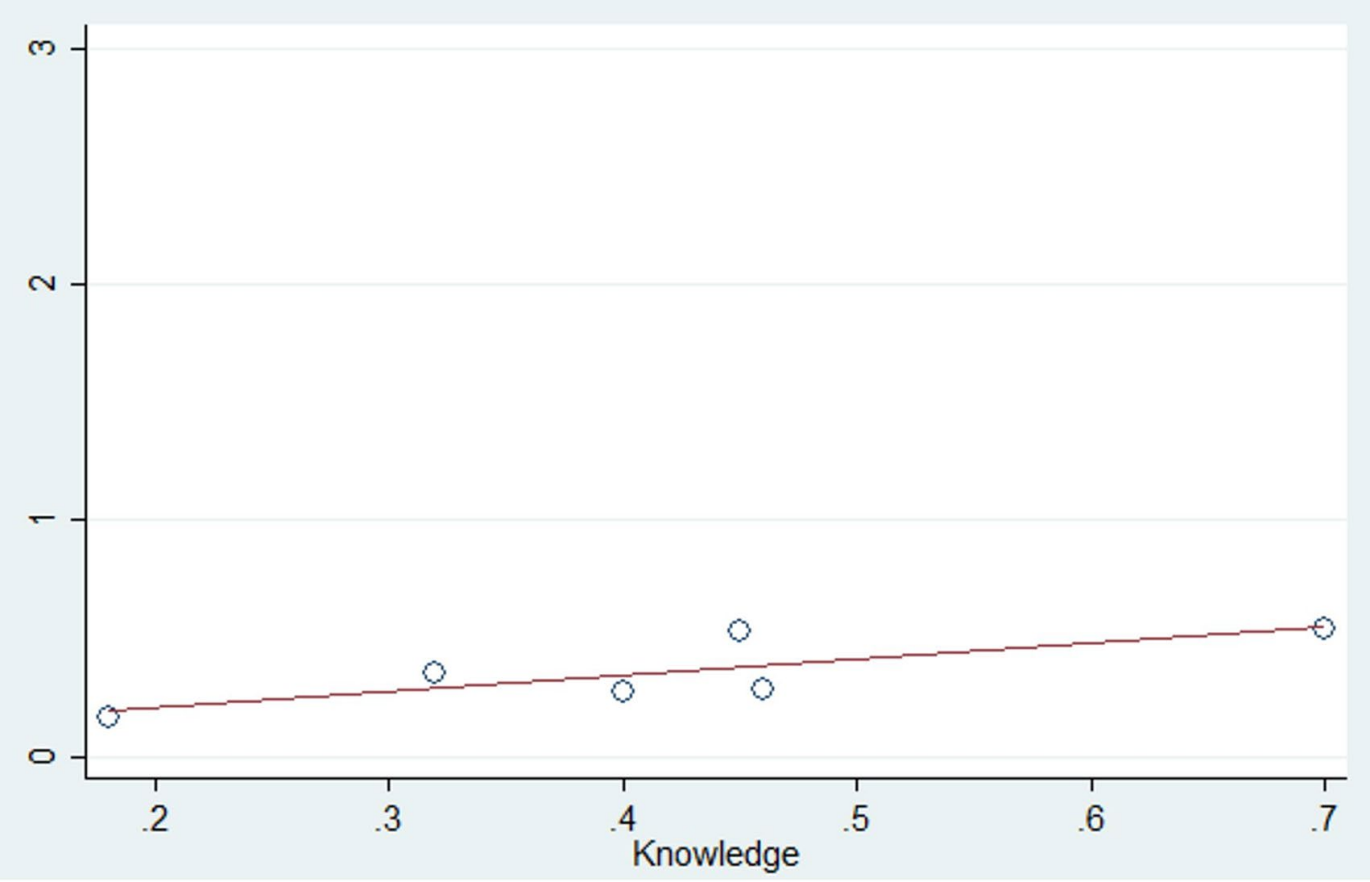
Bubble plot depicting the significant association between knowledge of HPV vaccine and vaccine uptake (*p* = 0.003).

Meta-regression analysis was conducted to assess factors affecting HPV vaccine uptake and attitude toward the vaccine. Covariates considered in the analysis included sample size, mean age, percentage of the sample that was female, employed, literate, knowledge of the HPV vaccine, and attitude toward the HPV vaccine. In the univariate meta-regression model, knowledge of the HPV vaccine uptake was found to be the only significant predictor for vaccine uptake (*p* = 0.003) (Table 2 and Figure 12).

**Table 2:** Meta-regression analysis for HPV vaccine uptake and attitude toward vaccine.

Predictor	Coefficient	Lower CI*	Upper CI	*p*-value
Predictors of vaccine uptake				
Sample size	0.0000821	−0.0006503	0.0008144	0.814
Mean age	−0.0082959	−0.0254163	0.0088245	0.296
Female	0.0022271	−0.0101223	0.0145765	0.706
Literate	0.002238	−0.0023272	0.0068032	0.300
Employed	0.0030751	−0.0008017	0.006952	0.108
Attitude	0.4648638	−0.5175919	1.447319	0.312
Knowledge	0.6897891	0.4038716	0.9757065	0.003
Predictors of attitude toward HPV vaccine				
Sample size	−0.0001731	−0.0009445	0.0005984	0.624
Mean age	0.0109225	−0.034089	0.0559339	0.584
Female	−0.0065467	−0.0232382	0.0101448	0.398
Literate	0.0151836	−0.0012357	0.0316029	0.064
Employed	−0.0010346	−0.0092556	0.0071864	0.775
Knowledge	−0.7173923	−1.986264	0.5514793	0.135

*CI, Confidence Interval; HPV, Human papillomavirus.

Similarly, in the univariate meta-regression model for attitude toward the HPV vaccine, covariates such as sample size, mean age, percentage of the sample that was female, employed, literate, and knowledge of the HPV vaccine were considered, but none of these factors were found to be significant predictors (Table 2).

## Discussion

This systematic review and meta-analysis aims to determine the estimated prevalence of HPV vaccine uptake in the South Asian region. Additionally, we assessed the proportion of participants who reported good knowledge and a positive attitude toward the vaccine. This meta-analysis is the first to report these metrics from the South Asian region and provides insights into effective strategies to increase HPV vaccine uptake.

Our study reveals a pooled prevalence of 8% (95% CI 1–21) based on data from over ten thousand participants. This estimate is significantly lower than those reported by similar meta-analyses focusing on other regions. Agimas et al. pooled data from 29 articles conducted in East Africa and reported a vaccine uptake of 35% (95% CI 26–45%) (38). Similarly, Addisu et al. pooled data from eight studies conducted in Ethiopia involving 3,936 participants and reported a vaccine uptake of 42.05% (95% CI 26.36–58.95) (39). Meanwhile, Bruni et al. conducted a pooled analysis of data from 64 countries and reported an uptake of 2.7% (95% CI 1.8–3.6), which aligns more closely with our findings (40). It’s worth noting that the global estimate of vaccine coverage is 77%, while a recent study reported a vaccine uptake of 62.8% in a low vaccination region of the United States (41, 42).

These findings underscore the alarming lack of progress in HPV vaccination and cervical cancer prevention in the South Asian region, particularly when compared to global efforts, developed countries, and even certain African regions (43). Sub-Saharan Africa, historically considered the center of HPV infection and cervical cancer, is home to 19 out of the top 20 countries with the greatest burden of cervical cancer (44). However, in recent years, 22 out of the 54 African countries have implemented national immunization programs (45). These efforts have undeniably provided a potential roadmap for South Asian countries to follow (46–48).

We report an estimated pooled prevalence for good knowledge of 41% (95% CI 28–55). This finding was in line with Addisu et al., who reported a prevalence of 55.12% (95% 30.8–79.4) (39). A meta-analysis reporting data from 58 Chinese studies reported lower knowledge (17.55, 95% CI 12.38–24.88) (49). While it is difficult to determine the exact cause for these discrepancies, one potential explanation is the relative recency of the studies included by our meta-analysis as well as by Addisu et al., compared to the Chinese study. Cervical cancer and HPV vaccination have gained increased attention globally in recent years (50–53). Another possible explanation is that several studies in our meta-analysis focused on healthcare professionals, who are generally expected to be more knowledgeable (54). However, only one such study could be included in the knowledge analysis. In the subgroup analysis of non-healthcare studies, 40% of participants still exhibited good knowledge. Despite this relatively high knowledge level, the low uptake of vaccines suggests that vaccine unavailability or high costs may be major factors contributing to the lack of vaccination (55–57).

Our study showed a significant association between the proportion of participants with good knowledge and the prevalence of vaccine uptake. These results are supported by previously published systematic reviews. Agimas et al. found good knowledge to be significantly associated with vaccine uptake (OR 1.6, 95% CI 1.43–1.8) (38). This was further corroborated by Addisu et al., who reported an odds ratio of 6.70 (95% CI 3.43, 13.07) (39). This association was also qualitatively reported by Kessels et al., who reviewed 25 studies, 20 of which were conducted in the United States (58). Greater knowledge of HPV and HPV vaccination likely enables individuals to make evidence-based decisions regarding vaccine uptake, as they become more receptive to national screening and vaccination efforts when aware of the consequences of HPV infection, as well as the relative efficacy, safety, and cost-effectiveness of vaccination (59, 60). Additionally, studies have shown that even just having heard of the HPV vaccine significantly increases vaccine uptake (61). These findings highlight the potential impact of mass awareness campaigns in significantly improving vaccine uptake, particularly in rural and low-literacy communities (62–65).

Our review indicates a positive attitude toward HPV vaccination in 56% of participants (95% CI 47–66). This is comparable to estimates of positive attitudes in Ethiopia (45.34%) and Germany (61.5%) (39, 66). Similar to knowledge, the South Asian region exhibits a comparable positive attitude toward vaccination compared to regions with higher vaccine uptake. This suggests that the limited vaccination is due to inadequate inclusion of HPV vaccination in national immunization programs (67–69). Although our study did not find attitude to be a significant predictor of vaccine uptake, substantial literature highlights a strong association between the two (70–73). In a meta-analysis conducted by Agimas et al., attitude was shown to be a significant predictor of uptake, with a reported OR of 2.54 (95% CI 2.13–3.03) (38). Similarly, Addisu et al. found a significant association with an OR of 2.04 (95% CI 1.51–2.74) (39). A Canadian focus group-based study revealed that poor knowledge and attitude were the most frequently observed barriers to vaccination (74). These results suggest that individuals with positive attitudes toward vaccines are more likely to have greater trust in vaccination programs. Furthermore, a longitudinal study conducted by Hofman et al. in the Netherlands demonstrated that a positive attitude was associated with increased trust in the vaccine and decreased ambivalence over time (75).

When comparing vaccine uptake across different countries, it is evident that Bhutan had a significantly higher uptake rate (99%) compared to India (6%), Pakistan (4%), and Bangladesh (3%). This difference can be attributed to Bhutan’s decision to implement a national HPV vaccination program in 2010, which achieved a coverage rate of 96.1% (76). Furthermore, Bhutan’s switch from health center-based to school-based immunization programs significantly improved coverage from 73 to 90% (76). Interestingly, data from Ethiopia, as reported by Addisu et al., also showed a relatively high uptake rate of 42%, mainly due to the implementation of school-based immunization programs (39). These findings suggest that school-based programs may be more effective in low-middle-income countries than health center programs. Bhutan’s success can also be attributed to various incentives employed, such as involving community leaders, providing token incentives like t-shirts, and offering financial incentives, which proved effective in their conservative and religious population (77). Other countries may achieve similar success by incorporating local cultural and traditional values, although this may pose challenges for culturally diverse countries like India and Pakistan.

It is important to interpret the findings of our systematic review, and meta-analysis needs to be interpreted with caution due to several limitations. Firstly, significant heterogeneity was observed in the pooled analysis. Despite undertaking subgroup and meta-regression analyses, we could not identify the source of heterogeneity. Secondly, despite conducting a thorough search, only 17 studies met the criteria for inclusion in the final analysis. The limited number of studies conducted may not comprehensively represent the wide range of HPV vaccine uptake in all South Asian nations, which could restrict the generalizability of the results. Thirdly, publication bias may exist due to the higher likelihood of studies with significant findings being published. We did a comprehensive search across many databases without any language limitations, it is possible that unpublished research or studies published in less widely available publications were inadvertently overlooked.

In conclusion, our study reveals that the prevalence of HPV vaccine uptake in South Asia is low (8%). The pooled prevalence of adequate knowledge and a favorable attitude toward the vaccine were 41 and 56%, respectively. In the univariate meta-regression model, knowledge of the HPV vaccine uptake was the only significant predictor of vaccine uptake. Based on these findings, we strongly recommend implementing awareness drives and social media campaigns to increase awareness about the HPV vaccine. Additionally, we suggest including the HPV vaccine in national immunization programs to prevent the region from lagging further behind the rest of the world in HPV vaccination rates and, ultimately, eradicate cervical cancer.

## Data Availability

The original contributions presented in the study are included in the article/supplementary material, further inquiries can be directed to the corresponding author.
